# Dysregulated expression of amino-acid and glucose transporters on circulating plasma cells in septic shock patients: a preliminary study

**DOI:** 10.1186/s40635-022-00472-5

**Published:** 2022-10-31

**Authors:** Margot Lepage, Morgane Gossez, Anne-Claire Lukaszewicz, Guillaume Monneret, Fabienne Venet

**Affiliations:** 1 Immunology Laboratory, Hôpital E. Herriot, Hospices Civils de Lyon, Edouard Herriot Hospital, 5 Place d’Arsonval, 69437 Lyon Cedex 03, France; 2 Centre International de Recherche en Infectiologie (CIRI), INSERM U1111, CNRS, UMR5308, Ecole Normale Supérieure de Lyon, Université Claude Bernard-Lyon 1, Lyon, France; 3 EA 7426 “Pathophysiology of Injury-Induced Immunosuppression” (Université Claude Bernard Lyon 1, Hospices Civils de Lyon, bioMérieux), Edouard Herriot Hospital, 69437 Lyon, France; 4 Anaesthesia and Critical Care Medicine Department, Hospices Civils de Lyon, Edouard Herriot Hospital, 69437 Lyon, France; 5 Joint Research Unit HCL-bioMérieux, Hôpital Edouard Herriot, 5 Place d’Arsonval, 69003 Lyon, France

## To the editor,

Sepsis, defined as a life-threatening organ dysfunction caused by a dysregulated host response to infection, perturbs immune homeostasis. In some patients, sepsis may lead to the development of a state of profound immunosuppression associated with increased susceptibility to secondary infections and mortality [1]. Mechanisms sustaining this immunosuppression are not fully understood.

Activation of specific metabolic pathways (aerobic glycolysis or oxidative phosphorylation) in immune cells is closely related to acquisition of effector versus regulatory functions. In sepsis, altered induction of aerobic glycolysis has recently emerged as a key mechanism involved in monocyte and T cell dysfunctions [1]. Elsewhere, amino-acid metabolism plays a central role in the regulation of B cell effector functions [2]. However, metabolic profile of circulating B cells remains poorly explored in sepsis, whereas B lymphocyte response is reported to be dysfunctional with decreased circulating number, marked plasmocytosis, reduced effector functionality and increased regulatory B cells [1, 3].

To investigate this aspect, in a preliminary study, we monitored overtime cell surface expressions of selected nutrient transporters related to glucose and amino-acid metabolisms in a cohort of nine septic shock patients and nine healthy volunteers (HV). GLUT1 (glucose importer), ASCT1 (neutral amino acids importer), and ASCT2 (mainly glutamine importer) expressions were evaluated by flow cytometry on circulating T cells, B cells, and plasma cells (Additional file 1: Fig. S1).

**Fig. 1:**
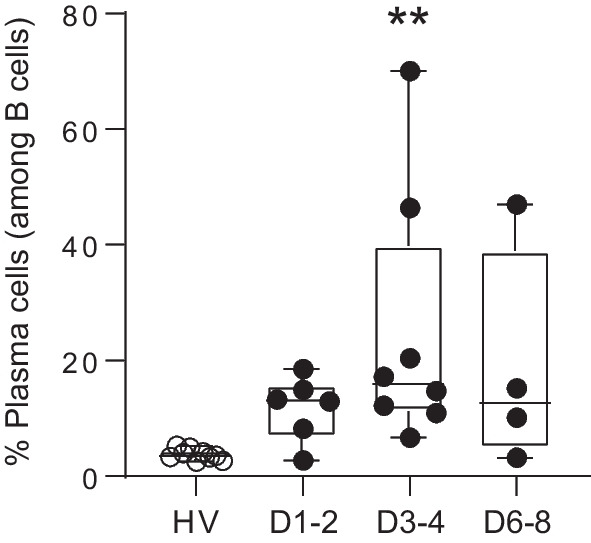
Plasma cells expansion in septic patients. Percentages of plasma cells (CD19^low^ FS^high^ cells—see Additional file 1: methods for gating strategy) among total CD19^+^ B lymphocytes were assessed by flow cytometry on peripheral whole blood in healthy volunteers (HV, *n* = 9) and septic patients (*n* = 9) sampled at day 1 or 2 (D1–2, *n* = 6), day 3 or 4 (D3–4, *n* = 8) and day 6, 7 or 8 (D6–8, *n* = 4) after the onset of septic shock. Data are represented as Tukey box-plots and individual values. **p < 0.005 compared to healthy volunteers. Non-parametric ANOVA test followed by post-hoc analysis with Dunn’s multiple comparisons tests

**Fig. 2:**
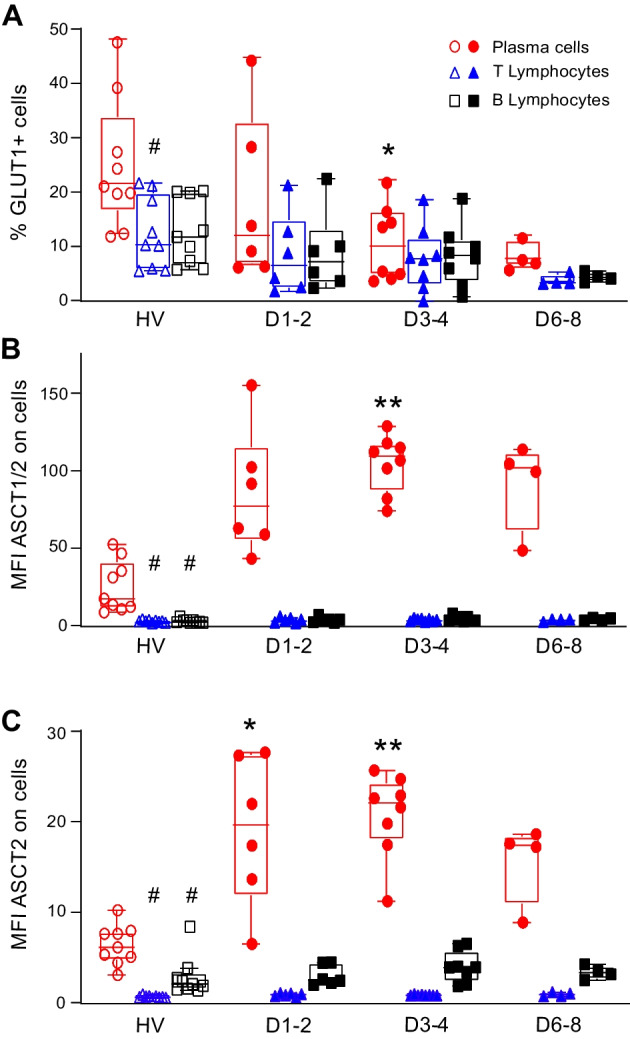
Nutrient transporters expressions on T cells, B cells and plasma cells during sepsis. Expressions of GLUT1 (percentage of positive cells - **Panel A**), ASCT1/2 (median fluorescence intensity - MFI, **Panel B**) and ASCT2 (MFI, **Panel C**) were assessed by flow cytometry on peripheral T cells (CD3^+^ lymphocytes, blue triangles), B cells (CD19^high^ FS^low^ lymphocytes, black squares) and plasma cells (CD19^low^ FS^high^ lymphocytes, red circles) from healthy volunteers (HV, *n* = 9) and septic patients (*n* = 9) at day 1 or 2 (D1–2, *n* = 6), day 3 or 4 (D3–4, *n* = 8) and day 6, 7 or 8 (D6–8, *n* = 4) after the onset of septic shock. Data are represented as Tukey box-plots and individual values. ^#^*p* < 0.05 compared with plasma cells in HV, nonparametric Mann–Whitney *U* test. *p < 0.05 and **p < 0.005 compared to identical cell population in HV. Non-parametric ANOVA test followed by post-hoc analysis with Dunn’s multiple comparisons tests

We first confirmed the occurrence of sepsis-induced immune alterations in patients with decreased monocyte HLA-DR expression, reduced CD4^+^ T cell count (Additional file 1: Table S1), and increased proportion of circulating plasma cells at D3–4 after sepsis (Fig. 1). As expected in HV, GLUT1, ASCT1, and ASCT2 expressions were higher on plasma cells compared to T and B lymphocytes (Fig. 2) [2]. When comparing patients and HV, we did not observe any difference in GLUT1, ASCT1, and ASCT2 expressions on T and B lymphocytes at any given timepoint (Fig. 2A). However, at D3–4, GLUT1 expression was significantly decreased on plasma cells from patients (*p* < 0.05, Fig. 2A), while ASCT1 and ASCT2 expressions were significantly increased (Fig. 2B, C).

Overall, we described the modified nutrient transporter expression profile of plasma cells with decreased glucose but increased amino-acid transporter expressions during sepsis-induced immunosuppression. As a shift from glycolytic to preferential oxidative metabolism of amino acids or fatty acids has been associated with acquisition of regulatory functions, we may hypothesize that the altered metabolic profile of plasma cells observed in the current study reflected their polarization toward regulatory functions. For example, a recent study in mice showed that *Plasmodium* infection induced an expansion of plasmablasts that over-expressed ASCT1 and ASCT2 mRNAs, which possessed regulatory functions through impairment of humoral immune response [4]. As the induction of regulatory plasma cells has been described in mice and human after sepsis [5], results from the current study suggested that metabolic alteration may represent a novel mechanism of regulatory plasma cell induction in sepsis. This now deserves to be further explored in dedicated pathophysiologic and mechanistic studies.

## Supplementary Information

**Additional file 1.** Online supplemental data.

## Data Availability

The data sets analysed during this study are available from the corresponding author upon request.
