# Supplementary material for: Dysregulated expression of amino-acid and glucose transporters on circulating plasma cells in septic shock patients: a preliminary study
Source: Intensive Care Med Exp. 2022 Oct 31;10:44. doi: 10.1186/s40635-022-00472-5 (PMC9618469; doi:10.1186/s40635-022-00472-5)
Supplement: Supplementary file 1 — Additional file 1. Online supplemental data. [file 40635_2022_472_MOESM1_ESM.docx]

**Online supplemental data**

**Dysregulated expression of amino-acid and glucose transporters on circulating plasma cells in septic shock patients: a preliminary study**

**Table S1. Clinical and biological data from septic shock patients**

| Patients’ clinical data (n=9) |  | |
| --- | --- | --- |
| Age (years) | 70 [60-72] | |
| Sex (male) | 7 (78%) | |
| Severity on admission |  | |
| SOFA score | 8 [6-9] | |
| SAPS II score | 54 [45-58] | |
| Lactates on admission (mM) | 2.8 [2.3-3.2] | |
| Type of admission |  | |
| Medical | 2 (22%) | |
| Surgery | 7 (78%) | |
| Localisation of Initial infection |  | |
| Intra-abdominal | 7 (78%) | |
| ORL | 1 (11%) | |
| Urinary tracts | 1 (11%) | |
| Microbiological documentation of initial infection | |  |
| Gram negative | 1 (11%) | |
| Gram positive | 6 (67%) | |
| Yeast | 2 (22%) | |
| Outcomes |  | |
| ICU-acquired infection | 0 (0%) | |
| ICU mortality | 2 (22%) | |
| Day 28 mortality | 2 (22%) | |
| Biological characteristics at day 3 (n=8) |  | |
| Lymphocytes (cells/mm^3^) | 855 [720-975] | |
| CD4+ T cells (cells/mm^3^) | 339 [296.25-530.75] | |
| mHLA-DR (AB/C) | 5793 [4025.75-10027.25] | |
| Treg cells (%) | 5.29 [4.82-5.85] | |

For clinical parameters, categorial data are presented as numbers of cases and percentages of the total population in brackets. Continuous data and biological parameters are presented as medians and interquartile ranges [Q1-Q3]. SAPS II (Simplified Acute Physiology Score II) and SOFA (Sequential Organ Failure Assessment) score were assessed on admission at ICU. Biological characteristics at day 3 are missing for one patient because he died before day 3. Normal range for number of anti-HLA-DR antibodies bound per monocyte (mHLA-DR AB/C) is 13 500 to 45 000 AB/C. Normal range for CD4+ T cells count is 336 to 1 126 cells/µL. Normal range for CD4^+^ CD25^high^ CD127^low^ regulatory T cells (Treg cells) is 4 to 10% of CD4+ T cells.

**Material and methods**

**Patients and healthy volunteers**

This study was conducted in the intensive care units (ICU) of Hospital Edouard Herriot (Lyon, France), as a part of a global study in sepsis induced immune dysfunctions (IMMUNOSEPSIS cohort). Nine septic shock patients were identified based on diagnostic criteria from the third International Consensus Definitions for Sepsis and Septic Shock (Sepsis-3). This project was approved by the Institutional Review Board for ethics (Comité de Protection des Personnes Sud-Est II, #IRB11236). This study is registered with the French Ministry of Research and Teaching (#DC-2008-509) and with the Commission Nationale de l’Informatique et des Libertés (CNIL). This study was registered at clinicaltrials.gov (NCT02803346). Non-opposition to inclusion in the study was recorded from each patient or next of kin. Exclusion criteria disqualified patients with presence of a pre-existent condition or treatment that could influence the immune status (e.g. HIV infection, use of immunosuppressive medication), hematological disease or a solid tumor within 5 years prior inclusion, extracorporeal circulation, pregnancy, aplasia, institutionalized patients and patients under the age of 18 years. Clinical data were collected for the nine patients including demographic characteristics, date and cause of admission to ICU, severity score at admission, status at day 28 after inclusion, type of infection and comorbidities (Table S1).

Concomitantly, peripheral blood from 9 healthy volunteers (HV, median age 59 years, interquartile range [56-62] years, 67% males) was provided by the Etablissement Français du Sang (EFS) from Lyon. According to the EFS standardized procedures and to provisions of the articles R.1243–49 and following ones of the French public health code, a written non-opposition to the use of donated blood for research purposes was obtained from HV and personal data were anonymized at the time of blood donation.

In patients, peripheral blood was collected at day 1-2 (D1-2, 6 samples collected), day 3– 4 (D3-4, 8 samples collected) and day 6-8 (D6-8, 4 samples collected) after the onset of septic shock. HV were sampled only once. The following immune parameters were evaluated in patients at D3-4 and in HV: lymphocytes and CD4^+^ T cells count, HLA-DR expression on monocytes and percentage of regulatory T cells CD4^+^ CD25^high^ CD127^low^. Expressions of nutrient transporters were evaluated in patients at each time point and in HV.

**Nutrient transporters expression**

Staining was performed on EDTA-anticoagulated fresh whole blood samples from septic shock patients recruited in our clinical protocol or from healthy donors. Red blood cells were lysed using VersaLyse lysing solution (Beckman Coulter) and washed with a buffer containing RPMI, sodium azide NaN_3_ and EDTA. Samples were then incubated with markers for 20 minutes at 37°C. Staining panel included lineage markers: PerCPVio700 labelled anti-CD3, VioGreen labelled anti-CD14 and PEVio770 labelled anti-CD19 (Reafinity, Myltenyi Biotec). In addition, cell surface expression of nutrient transporters (GLUT1, ASCT1 and ASCT2) was evaluated using RBD reagents (Metafora, Biosystems). These reagents are recombinant receptor binding site domain (RBD) targeting respectively GLUT1, ASCT1/ASCT2 or ASCT2 alone. These RBD are coupled directly with a fluorophore (GLUT1.RBD.eGFP) or coupled with a mouse Fc fragment to be used with a labelled anti-mouse secondary antibody (ASCT1/2.RBD.MouseFc or ASCT2.RBD.MouseFc + PE-labelled anti-mouse). Samples were then washed twice in sterile phosphate buffered saline solution (PBS, Eurobio). For secondary staining, samples were incubated with PE-labelled anti-mouse antibody for 30 min at 4°C and washed twice in PBS. Cells were then re-suspended in a buffer containing PBS, sodium azide NaN_3_, EDTA and foetal calf serum and were immediately acquired on flow cytometer.

Samples were run on a Navios flow cytometer (Beckman Coulter) and listmodes were subsequently analysed using Kaluza software (Version 2.1, Beckman Coulter). Gating strategy is presented in Figure S1. Briefly, percentages of plasma cells were assessed as CD19^low^ FS^high^ cells among all CD19^+^ cells. GLUT1 (glucose importer), ASCT1 (neutral amino acids importer) and ASCT2 (mainly glutamine importer) expressions were evaluated by flow cytometry on T, B and plasma cells. As GLUT1 presented a bimodal expression, results were expressed as the percentages of GLUT1-expressing cells among T lymphocytes (CD3^+^ cells), B lymphocytes (CD19^high^ FS^low^ cells) and plasma cells (CD19^low^ FS^high^ cells). As ASCT1 and ASCT2 presented a continuous expression, results were expressed as median fluorescence intensities (MFI) of ASCT1/2 and ASCT2 among each cell type.

**HLA-DR expression on monocytes**

Expression of HLA-DR on monocytes was performed to assess septic patients’ state of immunosuppression. Briefly, whole blood was stained with Quantibrite HLA-DR/Monocyte mixture (Quantibrite PE labelled anti-HLA-DR/PerCP-Cy5.5 labelled anti-CD14, Becton Dickinson). Samples were then lysed using the FACS Lysing solution (Becton Dickinson). HLA-DR expression on monocytes was measured on the surface of CD14^+^ cells as median fluorescence intensities (MFI) related to the entire cell population (as recommended by the manufacturer). These results were then expressed in antibody bound per cell (AB/C) thanks to calibrated PE-beads (BD Quantibrite PE Beads, Becton Dickinson). Normal range from the laboratory is 13 500 to 45 000 AB/C (1).

**Absolute CD4^+^ T cell count and regulatory T cells assessment**

Absolute CD4^+^ T cells count was assessed by an automated direct volumetric single-platform method on an Aquios CL flow cytometer (Beckman Coulter) as previously described (2). Whole blood is introduced in a microplate and then specific staining of leukocytes is accomplished by incubating whole blood with the monoclonal antibody reagent. It consists in a ready-to-use mix of antibodies currently used for routine analyses in the laboratory (panel Aquios Tetra-1, Beckman Coulter) containing: FITC labelled anti-CD45, PC5 labelled anti-CD3, PE labelled anti-CD4 and ECD labelled anti-CD8. Red blood cells are then removed by a no-wash erythrocyte lysis and remaining leukocytes are analysed by flow cytometry. Lymphocyte count relies on an algorithm including forward (FS) and side (SS) scatter characteristics, CD45 and electronic volume. CD4^+^ T cells count is assessed on a CD4/CD8 dot plot gated on CD3^+^ cells and lymphocytes.

For regulatory T cells (Treg) percentage assessment, whole blood was incubated at room temperature for 15 minutes in darkness in a tube coated with a layer of dried-down antibodies (DURAClone, Beckman Coulter). The panel used contained ECD labelled anti-CD4, PC5 labelled anti-CD25 and PE labelled anti-CD127. Then red blood cells were lysed by adding Optilyse C lysing solution (Beckman Coulter) and incubating at room temperature for 10 minutes in darkness. Samples were then washed once in sterile phosphate buffered saline solution (PBS, Eurobio) and acquired on a Navios flow cytometer (Beckman Coulter). Treg cells were gated as CD4^+^ CD25^high^ CD127^low^ cells.

**Statistical analyses**

Unless otherwise stated, results are presented as individual values, medians and 95% confidence intervals. A nonparametric Mann–Whitney U test was used to compare nutrient transporter expressions on plasma cells versus B or T cells in healthy volunteers. Non-parametric ANOVA test followed by post-hoc analyses with Dunn’s multiple comparisons tests were used to compare nutrient transporter expressions on any given cell subpopulation between healthy volunteers and septic patients at D1-D2, D3-D4 and D6-D8. Statistical analyses and graphics were performed on GraphPad Prism 5.03 (GraphPad). A p value inferior to 0.05 was considered statistically significant.

**Complementary Figures**

**
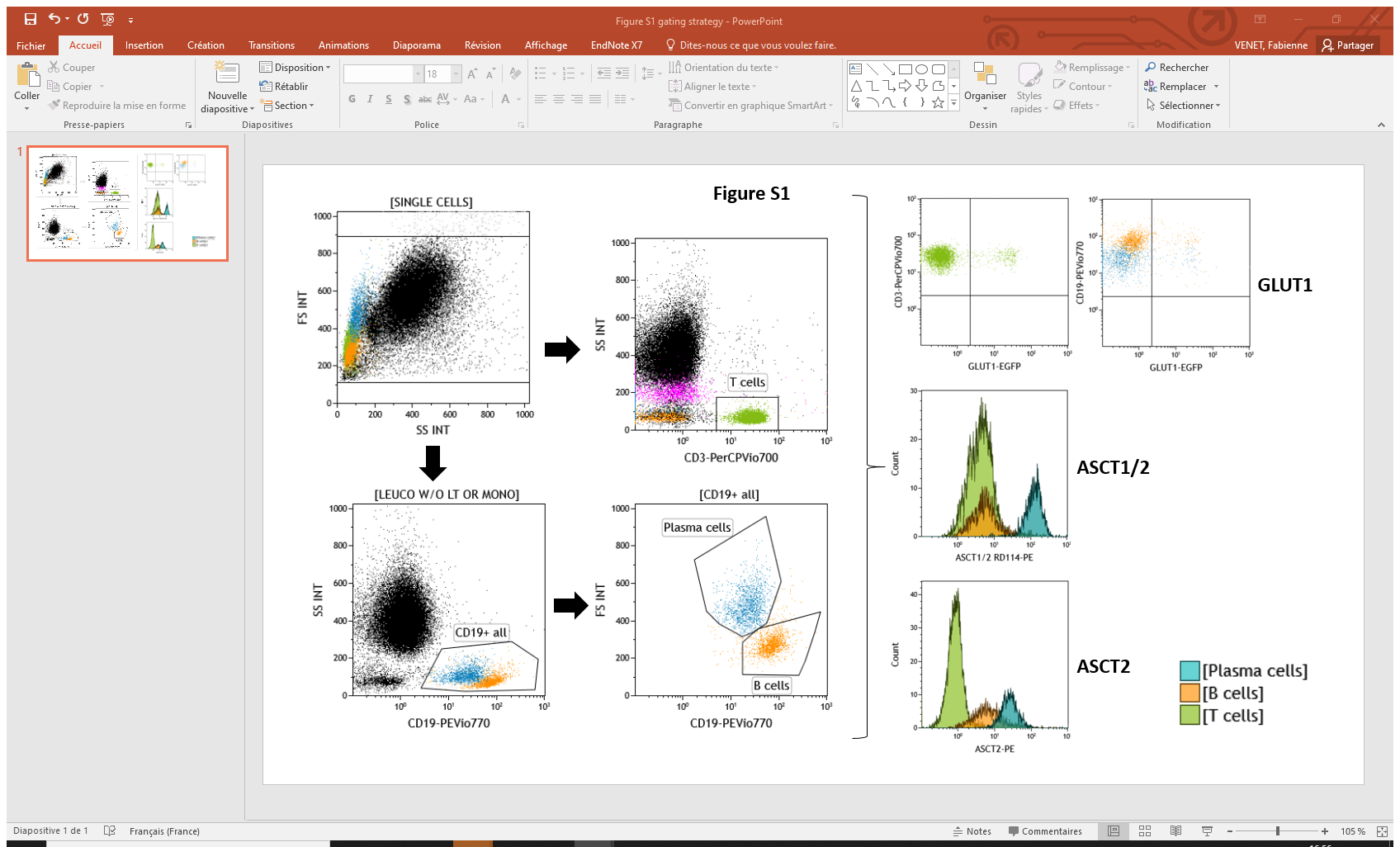
Figure S1. Illustrative example of gating strategy for a septic shock patient at D3-4**

CD3^+^ lymphocytes are identified on a dot plot histogram CD3/SSC after excluding doublets. Total CD19^+^ lymphocytes are identified on a dot plot histogram CD19/SSC after excluding doublets, CD3+ cells and CD14+ cells to enhance purity. On a dot plot histogram CD19/FSC gated on total CD19^+^ lymphocytes, B cells are identified as CD19^high^ FS^low^ cells and plasma cells as CD19^low^ FS^high^ cells. Expression of GLUT1 on each cell type is identified on dot plot histogram GLUT1/CD3 or GLUT1/CD19. Results are expressed as percentage of GLUT1 positive cells according to a common threshold settled to match all cell types in all patients and all healthy volunteers. Expression of ASCT1/2 and ASCT2 on each cell type is identified on monoparametric histograms and results are expressed as median fluorescence intensities (MFI) of the whole population.

**Complementary References**

1. Venet F, Textoris J, Blein S, Rol ML, Bodinier M, Canard B, Cortez P, Meunier B, Tan LK, Tipple C, Quemeneur L, Reynier F, Leissner P, Védrine C, Bouffard Y, Delwarde B, Martin O, Girardot T, Truc C, Griffiths AD, Moucadel V, Pachot A, Monneret G, Rimmelé T; REALISM study group (2022) Immune Profiling Demonstrates a Common Immune Signature of Delayed Acquired Immunodeficiency in Patients With Various Etiologies of Severe Injury. Crit Care Med 50:565-575
2. Gossez M, Malcus C, Demaret J, Frater J, Poitevin-Later F, Monneret G (2017) Evaluation of a novel automated volumetric flow cytometer for absolute CD4+ T lymphocyte quantitation. Cytometry B Clin Cytom 92:456-464
